# Dental Improvement

**Published:** 1854-02

**Authors:** 


DENTAL IMPROVEMENT.—We have received a circular, issued by Dr. Rickey, Surgeon Dentist of this city, in which he urges the value and importance of an improvement which he, jointly with Messrs. Crider & Williams of Ohio, claim to be the original discoverers, and to whom a joint patent was issued. From the history of the discovery it would appear that the priority belongs to our fellow-townsman, Dr. Rickey.

It is doubtless a valuable improvement, and should be adopted by all dentists, provided it embraces the advantages, as claimed by the circular, and of which, from the numerous attestations by the most eminent dentists of the Union, there is not a doubt. We extract from the circular the following as the fundamental features of the improvement:

1st, perfect adaptation; 2d, it allows the use of pure malleable gold, without the necessity of alloy to give it firmness; 3d, when from absorption of the alveolar process, it can be re-adapted with little difficulty; 4th, repairs do not injure the plate; 5, the teeth lighter but the strength even augmented; 6th, no possibility of accumulations to become offensive.

As Dr. Rickey has procured the right of one half the States, he is ready to make sale of office rights. Dr. Dunn, his agent, is about to visit this and others adjoining, will be empowered to make sale to those dentists who desire to keep pace with the improvements in their profession. We have been long acquainted with Dr. Dunn, and therefore bespeak for him that attention which he so well deserves.

